# Patient’s experiences with the care for juvenile idiopathic arthritis across Europe

**DOI:** 10.1186/s12969-018-0226-0

**Published:** 2018-02-08

**Authors:** E. H. Pieter. van Dijkhuizen, Tsipi Egert, Yona Egert, Wendy Costello, Casper Schoemaker, Marlous Fernhout, Mirjam Kepic, Alberto Martini, Silvia Scala, Ingrid Rotstein-Grein, Sebastiaan J. Vastert, Nico M. Wulffraat

**Affiliations:** 1 Paediatric Rheumatology, Wilhelmina Children’s Hospital, University Medical Centre Utrecht, Room KC.03.063.0, P.O. box 85090, 3508 AB Utrecht, The Netherlands; 2 Inbar Parent Association, Jerusalem, Israel; 3 iCAN Irish Children’s Arthritis Network, Dublin, Republic of Ireland; 4 Netherlands JIA patient/parent organization, Amsterdam, Netherlands; 5 Slovenian JIA patient organization, Ljubljana, Slovenia; 6 Paediatric Rheumatology, IRCCS G. Gaslini, Genoa, Italy; 7 Department of Pediatric Rheumatology, Hospital Pequeno Príncipe, Curitiba, Paraná, Brazil

**Keywords:** Patient participation, Care, Pediatric rheumatology, Europe

## Abstract

**Background:**

To assess the views of juvenile idiopathic arthritis (JIA) patients and their parents on the care and treatment they receive in referral pediatric rheumatology centers throughout Europe.

**Methods:**

In a collaboration between physicians and patient associations, a questionnaire was developed, covering various domains of JIA care, including demographics, diagnosis, referrals to various health care professionals, access to pain and fatigue management and support groups, information they received about the disease and awareness of and participation in research. The questionnaire was translated and distributed by parent associations and pediatric rheumatologists in 25 countries, 22 of which were European. After completion the replies were entered on the PRINTO website. Replies could either be entered directly by parents on the website or on paper. In these cases, the replies were scanned and emailed by local hospital staff to Utrecht where they were entered by I.R. in the database.

**Results:**

The survey was completed by 622 parents in 23 countries. The majority (66.7%) of patients were female, with median age 10–11 years at the completion of the questionnaire. Frequencies of self-reported JIA categories corresponded to literature. Some patients had never been referred to the ophthalmologist (22.8%) or physiotherapist (31.7%). Low rates of referral or access to fatigue (3.5%) or pain management teams (10.0%), age appropriate disease education (11.3%), special rehabilitation (13.7%) and support groups (20.1%) were observed. Many patients indicated they did not have contact details for urgent advice (35.9%) and did not receive information about immunizations (43.2%), research (55.6%) existence of transition of care clinics (89,2%) or financial support (89.7%). While on immunosuppressive drugs, about one half of patients did not receive information about immunizations, travelling, possible infections or how to deal with chickenpox or shingles.

**Conclusions:**

Low rates of referral to health care professionals may be due to children whose illness is well managed and who do not need additional support or information. Improvements are needed, especially in the areas of supportive care and information patients receive. It is also important to improve doctor patient communication between visits. Physicians can be instrumental in the setting up of support groups and increasing patients’ awareness of existing support. Suggestions are given to convey crucial pieces of information structurally and repeatedly to ensure, among other things, compliance.

**Electronic supplementary material:**

The online version of this article (10.1186/s12969-018-0226-0) contains supplementary material, which is available to authorized users.

## Background

There is much ongoing research in the realm of pediatric rheumatologic diseases in general, and juvenile idiopathic arthritis (JIA) specifically. The ultimate aim of this research is to provide benefits to the patients, in terms of better disease characterization, increased treatment efficacy and improved outcomes. To achieve this, a recent development has been to put more emphasis on the patient’s opinion in direct interaction between health care providers and patients advocacy groups and using so-called patient reported outcomes (PROs). [1] Indeed, the patient, as the most important stakeholder is pivotal in the evaluation of disease outcome. Active involvement of patients in evaluation of care and research is now a common goal for professional and patient organizations [2–4].

Consequently, the patients’ perspective with respect to their care and treatment is of utmost importance to identify areas in need of improvement. The patients’ and their parents’ views give insights into the organization of care for JIA patients in different countries, how the care is perceived by the patients and if it corresponds to existing guidelines and recommendations. Furthermore, we can find out if the patient has access to the range of services that exist or are needed. Additionally, investigation of the patients’ perspective brings to light if information given over to the patient is understood and retained. This is an important question, since it is known that during emotional stress little of the information received is remembered [5].

Armed with knowledge about these facets, specific points can be addressed to improve JIA care and to increase the patients’ awareness of available care where there is a discrepancy between the actual availability and the patients’ appreciation of it. Improved communication between physicians and patients can contribute to compliance (the parent understands what needs to be done and why), and patient satisfaction [6–10].

Therefore, the aim of this study was to survey a big panel of parents of JIA patients in a large number of mainly European countries regarding their perspectives on the care and treatment they receive.

## Methods

The study was performed by collaborators from the SHARE project, the Pediatric Rheumatology European Society (PReS) and the European Network for Children with Arthritis (ENCA). The survey was part of a larger project called SHARE conducted with the aim of optimizing and standardizing JIA care [11].

### Questionnaire description

The questions of the survey were grouped into four broad categories: (1) demographics and diagnosis; (2) organization of care; (3) social aspects; and (4) transition to adult care. In the demographics domain, specific questions included the age of the child at the time of completion of the questionnaire, sex and JIA category. In the questions about the organization of care, respondents were asked to give an indication of the waiting time between referral and the first visit, as well as of the interval between visits and the duration of the first and subsequent visits. Referrals to other health care professionals, such as physiotherapists, ophthalmologists and orthopedic surgeons, were also surveyed. Parents were asked if they received information about care and treatment, immunizations, schools and employers, nutritional needs, ongoing research, what to do in case of disease worsening, as well as if they knew about support groups (including financial support). The questionnaire covered information received upon starting biological medications as well. Specific attention was paid to visits to the ophthalmologist and the frequency as well as the location of these visits. A group of questions was dedicated to research: Parents were asked to indicate if they were aware of registries and drug trials and if their child participated in any of these. Access to different health care services was surveyed as well, such as pain management, fatigue management and rehabilitation.

The social domain of the questionnaire covered questions pertaining to awareness of the existence of and access to support groups, as well as if the school the child was attending had been contacted by a member of the rheumatology team. Finally, the domain pertaining to transition care asked if parents and their children received information about the transitioning to adult care and addressed specific adolescent issues, such as alcohol, drugs, sexual health and pregnancies. The full questionnaire is included in the supplementary material.

The questionnaire was distributed using two main routes: Through pediatric rheumatologists who were members of the pediatric rheumatology international trials organization (PRINTO) in 25 countries, and by the individual parent associations in each country, thus possibly including JIA patients treated by physicians other than pediatric rheumatologists. The replies were not monitored for missing values or inconsistencies. The questionnaire was completed anonymously by patients with JIA according to international league against rheumatism (ILAR) criteria.

Review by the local ethics committees was not required, since this research was initiated by the patients themselves and included a questionnaire only.

### Statistical analysis

The anonymized survey results were summarized by calculating the median, 1st quartile and 3rd quartile for continuous data and the number and frequency for categorical data. Countries were categorized into western and non-western countries (Additional file 1: Table S1) and differences between these regions were tested using Pearson’s χ^2^-test or Fisher’s exact test for categorical data and the Mann-Whitney U test for continuous data.

Statistical analysis was carried out using R 3.3.2 (R Foundation for statistical computing, Vienna, Austria).

## Results

### General demographics

The questionnaire was completed by 622 patients in 23 countries (420 patients, 67.5%, in 15 western countries and 202 patients, 32.5%, in 8 non-western countries; Table 1; Additional file 1: Tables S1 and S2). No responses were obtained from centers in two countries. The majority of patients were female (66.7%) and the median (1st quartile-3rd quartile) age at the time of completion of the questionnaire was 10 years (7–15) in western countries and 11 years (8–15) in non-western countries. There were no differences in sex or age distribution between western and non-western countries (Table 1). The median age at diagnosis was equal in both western and non-western countries (5 years; Table 1). The most frequent self-reported JIA categories were oligoarthritis (25.2% and 30.7% in western and non-western countries, respectively) and rheumatoid factor (RF) negative polyarthritis (24.5% and 20.3% in western and non-western countries, respectively). There were differences mainly in the proportion of patients indicating undifferentiated arthritis and an unknown category of JIA between western and non-western countries (Table 1).

**Table 1:** Patient demographics

	Western countries	Non-western countries	*P*-value^a^
*N*	420	202	
Female, *n* (%)	286 (68.1%)	129 (63.9%)	0.34
Age at completion, median [1st quartile, 3rd quartile]	10 [7, 15]	11 [8, 15]	0.50
Age at diagnosis, median [1st quartile, 3rd quartile]	5 [2, 9]	5 [3, 10]	0.04
Self-reported JIA category, *n* (%)			
Systemic	57 (13.6%)	23 (11.4%)	0.53
Oligoarthritis	106 (25.2%)	62 (30.7%)	0.18
Polyarthritis RF -	103 (24.5%)	41 (20.3%)	0.29
Polyarthritis RF +	46 (11.0%)	14 (6.9%)	0.15
Psoriatic	21 (5.0%)	5 (2.5%)	0.21
ERA	25 (6.0%)	12 (5.9%)	> 0.99
Undifferentiated	19 (4.5%)	1 (0.5%)	0.02
Don’t know	43 (10.2%)	44 (21.8%)	0.0002

^a^P-value of the test of differences between western and non-western countries. For categorical data Pearson’s χ^2^-test was used; for continuous data the Mann-Whitney U test

Abbreviations: *ERA* enthesitis-related arthritis, *JIA* juvenile idiopathic arthritis, *RF* rheumatoid factor

### Referrals and intervals between visits

The median (1st quartile-3rd quartile) time between the referral and the first visit was shorter in western countries (14 [5–30] days) when compared to non-western countries 21 7–61] days; *p* = 0.001). The median time interval between two visits was equal for both regions (3 months). In non-western countries, 155 respondents (76.7%) replied that more than 45 min were allotted to the first visit, compared to 238 respondents (56.7%; *p* < 0.0001) in western countries. Likewise, more respondents in non-western countries indicated that more than 25 min were allotted to follow up visits (*n =* 148, 73.3%, compared to *n =* 185, 44.0%; *p* < 0.0001).

### Ophthalmology, physiotherapy, and multidisciplinary care

The majority of respondents had been referred to the ophthalmologist at least once (Table 2). Almost 4 out of 10 respondents were being screened at least every 3 months (*n =* 244, 39.2%) and 46.3% (*n =* 288) were being screened every 6 months. The data collected did not include information about how many of the patients had uveitis at the time of the survey. There were no differences between western and non-western countries.

**Table 2:** Referrals and access to supportive care

	Western countries	Non-western countries	*P*-value^a^
*N*	420	202	
Referrals to:			
Ophthalmologist	327 (77.9%)	153 (75.7%)	0.63
Physiotherapist	270 (64.3%)	155 (76.7%)	0.002
Occupational therapist	93 (22.1%)	8 (4.0%)	< 0.0001
Social worker	44 (10.5%)	1 (0.5%)	< 0.0001
Psychologist	95 (22.6%)	19 (9.4%)	0.0001
Orthodontist	79 (18.8%)	60 (29.7%)	0.003
Indicated access to:			
Pain management	54 (12.9%)	8 (4.0%)	0.0009
Fatigue management	16 (3.8%)	6 (3.0%)	0.77
Support group	108 (25.7%)	17 (8.4%)	< 0.0001
Age appropriate disease education	49 (11.7%)	21 (10.4%)	0.74
Special rehabilitation	36 (8.6%)	49 (24.3%)	< 0.0001
Counselling and psychological support	177 (42.1%)	35 (17.3%)	< 0.0001

^a^*P*-value of the test of differences between western and non-western countries, using Pearson’s χ^2^-test

More patients in non-western countries had visited the physiotherapist compared to western patients (Table 2). Most patients had never been referred to any of the following health professionals: occupational therapist, social worker, psychologist or orthodontist (Table 2). Moreover, only a minority of patients indicated having access to pain and fatigue management, support groups, age appropriate disease education and special rehabilitation. A minority in both regions indicated having access to counselling and psychological support (Table 2). The majority of patients from western countries (*n =* 348, 82.9%) were aware of the existence of patients groups, forums or network meetings, compared to a minority of non-westerners (*n =* 45, 22.3%; *p* < 0.0001). Few respondents (*n* = 67, 10,8%) indicated they were aware of the presence of a transition of care clinic in their center, This was similar is western and non-western countries.

### Emergency information and maintaining contact with doctor between visits

The majority of patients received information about ongoing care and treatment. There was an indication that non-western patients received more often information about what to do in case of worsening symptoms (Table 3). Fewer westerners than non-westerners indicated having received contact details for urgent advice. Overall, more non-westerners than westerners could contact their pediatric center by phone and had a telephone help line or e-mail contact available. Western patients experienced longer waits for a response more frequently compared to non-western patients (Table 3).

**Table 3:** Patient-physician communication

	Western countries	Non-western countries	*P*-value^a^
*N*	420	202	
Received information about:			
Ongoing care and treatment	372 (88.6%)	187 (92.6%)	0.16
What to do in case of worsening symptoms	316 (75.2%)	168 (83.2%)	0.03
Contact details urgent advice	251 (59.8%)	148 (73.3%)	0.001
Immunizations	220 (52.4%)	133 (65.8%)	0.002
Support groups	136 (32.4%)	17 (8.4%)	< 0.0001
Financial support	39 (9.3%)	25 (12.4%)	0.30
School/employers	87 (20.7%)	84 (41.6%)	< 0.0001
Healthy eating	59 (14.0%)	62 (30.7%)	< 0.0001
Research	166 (39.5%)	110 (54.5%)	0.0006
Appropriate advice with respect to medication:			
Immunizations/vaccines	251 (59.8%)	150 (74.3%)	0.0006
Travel advice	130 (31.0%)	106 (52.5%)	< 0.0001
Possible infections	207 (49.3%)	135 (66.8%)	< 0.0001
How to deal with chickenpox or shingles	96 (22.9%)	106 (52.5%)	< 0.0001
Means of communication:			
Contact paediatric centre by phone	195 (46.4%)	144 (71.3%)	< 0.0001
Telephone help line	169 (40.2%)	110 (54.5%)	0.001
E-mail	212 (50.5%)	147 (72.8%)	< 0.0001
Waiting time reply for non-urgent questions:			
< 24 h	128 (30.5%)	67 (33.2%)	0.56
> 24 h and < 48 h	140 (33.3%)	62 (30.7%)	0.57
> 48 h	69 (16.4%)	14 (6.9%)	0.002

^a^*P*-value of the test of differences between western and non-western countries, using Pearson’s χ^2^-test

### Doctors as a source of specific and general JIA information, including information about immunizations

Many patients reported that they did not get information about immunizations, support groups, financial support, school or employers, and healthy eating (> 40%). Overall, patients in western countries received more frequently information about support groups, whereas patients in non-western countries received more frequently information about immunizations, school or employers and healthy eating (Table 3). With respect to information received regarding immunosuppressive drugs, one third to one half of patients did not receive information about immunizations and possible infections, while more than half did not receive travel advice or guidance on how to deal with chickenpox or shingles.

Overall, non-westerners got more information than westerners (Table 3).

### Information about trials and research

About one half of patients received information about research (Table 3). Slightly fewer respondents were aware that information was being collected about treatment and side effects (*n =* 152, 36.2% in western countries and *n =* 104, 51.5% in non-western countries; *p* = 0.0004). More western than non-western patients had been approached to enter a trial (*n =* 169, 40.2% vs. *n =* 29, 14.4%; *p* < 0.0001). Conversely, non-western patients participated more frequently in a registry than western patients (*n =* 75, 37.1% vs. *n =* 93, 22.1%; *p* = 0.0001).

## Discussion

The data from this survey provided a unique view regarding care, opinions, experiences and information received by 622 JIA patients and their parents, living in 21 European countries, Israel and Turkey (Additional file 1: Table S1). As such this data collection is unique and relevant for an analysis of the perceived quality of care.

### Patient’s knowledge on their illness

JIA patients seemed to be well aware of their disease category. The frequencies of the categories in our sample corresponded to reports in literature,[12] with the exception of a slightly lower frequency of oligoarthritis and a slightly higher frequency of polyarticular RF positive arthritis in western countries. This may be due to a misclassification of extended oligoarticular patients as polyarticular patients. Furthermore, the frequency of undifferentiated arthritis in our sample was much lower than the frequency reported in literature. These patients might have reported their category as unknown.

### Ophthalmologic screening, physiotherapy and transition of care

Frequent ophthalmologic screenings are important for JIA patients, especially for those with positive antinuclear antibodies (ANA) [13]. In our sample, more than one in five patients indicated they did not visit the ophthalmologist. When factoring in the 80 systemic JIA patients (12.9%), who do not need regular ophthalmologic screening, approximately 10% additional patients should be receiving regular screening, but indicated they did not.

Similarly, referrals to the physiotherapist were reported by a low percentage of patients. Physiotherapy is important because children with JIA show markedly reduced levels of physical activity and energy expenditure, regardless of disease activity [14–17]. These reduced activity levels in turn have detrimental effects on functional ability, bone mass and strength, and quality of life [17, 18] Moreover, pre-school aged children with JIA frequently demonstrate severe to moderate delays in the achievement of milestones [19]. Functional therapies including physiotherapy may help to overcome these detrimental effects. A number of studies have shown the potential of physical therapy as well as its safety in the non-active phase of the disease [17, 20–22].

The percentage of respondents that was aware of transition of care clinics was remarkably low, both in east and west. This was also true for parents of JIA patients over 15 years of age. This indicates that transition programs seem to start well after this age, despite the recommendation to start this process early.

### Referrals to other professionals and various support systems

The rate of referrals to other professionals, such as occupational therapists, social workers and psychologists was very low in both western and non-western countries. On top of that, many respondents indicated to have no access to pain and fatigue management, support groups, age appropriate disease education and psychological support. Even though this kind of support might actually be available, these answers implied that patients were not aware of their existence. Given that pain and fatigue burden the patients’ daily lives considerably [16, 23] and that many JIA patients experience difficulties at school or work, [24–28] these forms of supportive care are of utmost importance and should be made available widely.

Parent associations help to educate parents about treatments and compliance. They give information that provides practical, financial and social support to children and their families. They also support families to help their child develop happily and normally. Yet, our data indicated that parent associations do not exist in all countries (Table 2), or that, even when they do exist, doctors do not refer parents to them often enough (Table 3). Physicians can be instrumental in the creation of support groups, by helping patients get in touch with each other. On the other hand, if these support systems are already available, physicians are pivotal in making patients aware of their existence.

### Information about ongoing care and treatment

Much improvement is needed with respect to informing the patient: A sizable minority of 20–25% of patients did not know what to do in case of worsening symptoms.; And only 40–50% of patients had received information about immunizations. It might be that physicians provided insufficient information to patients, [29–31] or patients may not have understood the explanation given by the physician, possibly due to the use of medical terminology [6]. Moreover, many patients indicated not having a telephone help line web-portal or e-mail contact at their disposal.

It is well known that information provided by the clinician is not always remembered by patients. This contributes to a knowledge gap that stems from emotional overload that parents experience during the first visits when much of the information is given, added to the difficulty of assimilating large amounts of new and unfamiliar information under stress. Indeed, it was previously shown that information retention is in the range of only 50–90% [7, 32, 33]. The information retention problem is worse in case of emotional stress, such as after a first visit to the oncologist [5]. Conversely, more information is remembered after follow up visits in comparison with first visits [32, 33]. The information problem is important, as effective physician-patient communication may lead to better health outcomes, increased trust and higher satisfaction [6–10]. Therefore, we suggest the following improvements to the communication:

1. The physician should repeat crucial information (including booklets and websites) starting about 3 months after the initial diagnosis and continuing up to at least 1 year, making sure that the information is understood;
2. Each visit, a short summary should be available to the patient, containing at least the following information: full name of the condition from which the child suffers, including category and ANA status; general state of health found during the visit; list of medications and dosages the child should be taking; list of laboratory and other tests that need to be performed (including tuberculosis screening when necessary). The information should include reminders for visits to the ophthalmologist and any other health care professional and a phone number or an e-mail address through which the doctor can be contacted in case of need. Early experience with providing such information by unrestricted access to medical files through a web portal indicates that only few patients wish to use this regularly;
3. Online access to medical files using portal should be encouraged. Medical files should contain a section where the goal of therapies as phrased by patients is documented.

These suggestions are in line with recent research, showing that the ability to recall information is improved by using simple language, [34] written information, [35] and the introduction of new information at intervals, when it becomes more relevant [36].

### Differences between western and non-western countries

Overall, patients in western countries had access to supportive care more frequently, such as occupational therapists, social workers, psychologists, pain management and support groups. On the other hand, patients in non-western countries more frequently indicated referrals to the physiotherapist and the orthodontist as well as access to special rehabilitation. In between contact with centers was more frequently available for eastern country residents and they reported shorter response times, compared to western patients. Furthermore, non-western patients indicated having received more advice with respect to medication.

### Research

We examined the patients’ awareness of research, since participation in trials places them as a partner in the quest for better treatment of JIA and encourages them to share their experiences with treatment. Moreover, trials and registrations form an integral part of the health care in many third line centers. However, the results indicated that not many patients were aware of on-going research, let alone involved in the planning and performing of trials.

### Limitations

When interpreting these data, one should take into account that the questionnaire was distributed primarily among pediatric rheumatologists, who are members of the networks of PRES, PRINTO and patient organizations. Therefore, the respondents form a biased group of JIA patients who were likely to receive the best possible care per country. Consequently, the actual situation might be less favorable than the responses indicated. This may be the case especially in non-western countries, where many JIA patients may be treated by physicians other than pediatric rheumatologists. Furthermore, the data presented in this study exclusively represented the respondents’ view. The answers were not verified, for example to check whether support groups of transition of care clinics truly did not exist in case respondents indicated they did not have access to them. Therefore, in our analysis we focused on patients’ awareness of supportive care, rather than its existence, as the effectiveness of supportive care is diminished when patients are unaware of its existence. Moreover, even though the questionnaire was anonymous, many physician’s handed the questionnaire to the patients and received the completed form from them. This might have influenced the answers. Finally, given the big number of hypotheses tested in this manuscript, *p*-values in the range from 0.01 to 0.05 should be interpreted as providing only weak evidence [37].

## Conclusion

In conclusion, the data provided a unique view on JIA patients’ and their parents’ perspectives regarding the care they receive. Improvements are needed mainly in the areas of supportive care and the accessibility of information to patients. The rate of referral to ophthalmologists and physiotherapists could be increased too. Suggestions have been made to convey crucial information structurally and repeatedly and to increase the awareness of the existence of supportive care, such as pain and fatigue management as well as support groups, or to start new initiatives where these are lacking.

We conclude that a sizable knowledge gap still exists in many aspects of care. Engagement of patient groups is important and a step towards active patient participation in evaluating received care and setting joint research agendas to improve this.

## Additional file

Additional file 1: Table S1. Number of questionnaires per country and division into regions. **Table S2.** SHARE PARENT SURVEY: Patient Personal Information and Diagnosis. (DOCX 24 kb)

## Abbreviations

ANA: antinuclear antibodies (ANA
ENCA: European Network for Children with Arthritis
JIA: Juvenile Idiopathic Arthritis
PRES: Pediatric Rheumatology European Society
PRINTO: Pediatric Rheumatology International Trials Organisation
PRO: Patient Reported Outcomes
SHARE: Single Hub and Access point for pediatric Rheumatology Europe
