# Supplementary material for: Patient’s experiences with the care for juvenile idiopathic arthritis across Europe
Source: Pediatr Rheumatol Online J. 2018 Feb 8;16:10. doi: 10.1186/s12969-018-0226-0 (PMC5806356; doi:10.1186/s12969-018-0226-0)
Supplement: Supplementary file 1 — Number of questionnaires per country and division into regions. Table S2. SHARE PARENT SURVEY: Patient Personal Information and Diagnosis. (DOCX 24 kb) [file 12969_2018_226_MOESM1_ESM.docx]

| **Supplementary Table 1. Number of questionnaires per country and division into regions** | | |
| --- | --- | --- |
| Country | Region | *N* |
| Albania | Non-western | 1 (0.2%) |
| Austria | Western | 1 (0.2%) |
| Belgium | Western | 16 (2.6%) |
| Czech Republic | Non-western | 21 (3.4%) |
| Denmark | Western | 34 (5.5%) |
| France | Western | 61 (9.9%) |
| Germany | Western | 28 (4.5%) |
| Greece | Non-western | 30 (4.8%) |
| Ireland | Western | 26 (4.2%) |
| Israel | Western | 29 (4.7%) |
| Italy | Western | 32 (5.2%) |
| Latvia | Non-western | 21 (3.4%) |
| Lithuania | Non-western | 1 (0.2%) |
| Morocco | Non-western | 1 (0.2%) |
| The Netherlands | Western | 100 (16.2%) |
| Portugal | Western | 5 (0.8%) |
| Serbia | Non-western | 5 (0.8%) |
| Slovakia | Non-western | 33 (5.3%) |
| Slovenia | Non-western | 17 (2.7%) |
| Spain | Western | 20 (3.2%) |
| Sweden | Western | 1 (0.2%) |
| Switzerland | Western | 1 (0.2%) |
| Turkey | Non-western | 102 (16.5%) |
| United Kingdom | Western | 31 (5.0%) |
| United States | Western | 2 (0.3%) |
| Total |  | 619 |

**Supplementary Table 2.**

**SHARE PARENT SURVEY: Patient Personal Information and Diagnosis**

Country of birth

Gender: (Male) (Female)

Age:

Age at diagnosis:

Person completing the questionnaire:

- Mother
- Father
- Child
- Legal guardian

1. Please indicate the JIA category of your child

- Systemic Arthritis
- Oligoarticular Arthritis
- Polyarticular Arthritis (Rheumatoid factor negative)
- Polyarticular Arthritis (Rheumatoid factor positive)
- Psoriatic Arthritis
- Enthesitis Related Arthritis
- Undifferentiated Arthritis

2. Was your child referred to a paediatric rheumatologist within 6 weeks of reporting the initial

symptoms to your doctor?

- yes
- no, but within ____ weeks
- no (because there isn’t one)

3. How long was it between referral to the paediatric rheumatologist and the first clinic

appointment?

- ____days
- ____weeks
- ____months

4. Did you have the initial rheumatology consultation with a paediatric rheumatology

consultant?

- yes
- no, but with
- a paediatrician who has an interest in rheumatology
- a rheumatologist for adults
- a general practitioner
- an orthopaedic surgeon

5. How long did your first rheumatology consultation last?

- less than 45 minutes
- more than 45 minutes

6. At that initial consultation did you receive information about (tick all that apply)

- what to do in case of worsening symptoms
- contact details for urgent advice
- types of treatment
- members of the paediatric rheumatology multidisciplinary team
- national or local support groups/helplines
- details of financial support available
- school/employers
- healthy eating / nutritional needs
- immunizations/ vaccines
- other (please specify)

**Subsequent Care**

1. How long approximately is the interval between appointments with your paediatric

rheumatologist?

- ___ weeks
- ___ months

2. How long on average is your consultation time?

- less than 25 minutes
- more than 25 minutes

3. During follow up consultations have you ever received information about (tick all that apply)

- ongoing care and treatment
- what to do in case of worsening symptoms
- contact details for urgent advice
- immunizations e.g flu vaccination
- research
- national or local support groups/helplines
- details of financial support available
- school/employers
- healthy eating / nutritional needs
- other (please specify)

4. How far is it from your home to the (paediatric) rheumatology centre (hospital)

- ____ km
- ____ miles
- ____ hours

5. Has your child ever had a referral from a paediatric rheumatologist to a (tick all that apply )

- physiotherapist
- occupational therapist
- podiatrist/orthopaedic shoemaker
- specialist nurse
- social worker
- psychologist
- ophthalmologist
- orthopaedic surgeon
- orthodontist
- paediatric surgery ward
- none of the above

6. Can you contact the paediatric rheumatology centre by telephone (24 hour service) if need be?

- yes
- no
- I don’t know

7. How long does it take to receive a reply from your rheumatology centre for a non-urgent

problem?

- less than 24 hours
- within 48 hours
- more than 48 hours

8. To whom are the doctor´s notes/ medical reports copied?

- you/your family
- your referring doctor
- I don’t know

9. How is your child´s disease activity assessed? (tick all that apply)

- physical examination
- additional tests Ultrasound /MRI
- questionnaire

10. If your child is taking immunosuppressive agents (e.g. corticosteroids, methotrexate, biological

agents); is there appropriate advice (verbal and written) available on the following? (tick all that

apply)

- immunizations/vaccines
- travel advice
- possible infections
- how to deal with chickenpox or shingles
- not applicable

11. Is your child´s pain level appropriately assessed frequently? (eg. Visual Analogue Scale =VAS on a scale from 0-10 or smileys ).

- yes
- no
- I don’t know

12. Is your child screened and managed by an ophthalmologist experienced in paediatric uveitis?

- yes
- no
- I don’t know

13. How often is your child screened by an ophthalmologist?

- less than 3 months
- every 3 months
- every 6 months

14. Is your ophthalmology screening at the same location and time as your rheumatology review

appointment?

Same location

- yes
- no
- not applicable

Same time

- yes
- no
- not applicable

15. Do you have access to specialist surgery, carried out by specialists trained in JIA? (tick all that

apply)

- paediatric orthopaedic surgery
- maxillofacial surgery
- hand surgery
- I don’t know
- other (please specify)

16. If you/your child needs biologic drugs, can you/your child access them?

- yes
- no
- I don’t know

17. Are you aware of information being collected about your/your child’s treatment and side effects, either on a national or international level by means of a drug registry?

- yes
- no

If yes, is your child participating in one of these registries?

- yes
- no

18. Are you aware of any research being carried out in paediatric rheumatology?

In your country

- yes
- no

Abroad

- yes
- no

19. Have you ever been approached to enter a paediatric rheumatology trial?

- yes
- no

20. Does your child participate in a national and/or international drug registry (biologic

pharmacovigilance/efficacy studies, e.g. MTX or biologicals)?

- yes
- no
- I don’t know

21. Do you have access to the following services (tick all that apply)

- telephone help line
- email
- pain management
- fatigue management
- age appropriated disease education
- special rehabilitation
- transition care
- support group , in or out of the hospital

22. Do you have access to occupational therapy aids/equipment if needed? (e.g. splints, wheelchair, crutches, laptop)

- yes
- no
- I don’t know

23. Has your child ever required an overnight stay in hospital because of their arthritis treatment or condition?

- yes
- no

If yes, did you have the opportunity to stay with her/him?

- yes
- no

**Social aspect**

1. Has a member of the rheumatology team or local doctor contacted your child´s nursery /school regarding his/her condition?

- yes
- no
- I don’t know

2. Did you receive information about social/legal rights e.g. entitlements?

- yes
- no
- not applicable

3. Are you aware of local groups/forums/network meetings outside the medical setting, in order to share your experiences with others?

- yes
- no

4. Do you have access to any professional support for your child and/or family e.g. counselling,

psychological support?

- yes
- no
- I don’t know

**Transition – the period of time a child moves from paediatric to adult services**

Is this applicable to your child for his/her age?

- yes
- no

If applicable to your child please answer the following questions.

1. Are you aware of written information and advice being provided about (tick all that apply):

- alcohol
- drugs
- sexual health
- relationships
- pregnancy
- education/jobs
- studies
- social welfare entitlements

2. Have you ever received information about transition from paediatric to adult care?

- yes
- no

If yes, who belongs to the transition team? (tick all that apply)

- paediatric rheumatologist
- rheumatologist
- nurse
- psychologist
- physiotherapist
- social worker
- occupational therapist

3. At what age did you first start to discuss transition to adult services?

- At the age of _____
